# Delayed haemolysis after treatment with intravenous artesunate in patients with severe malaria in India

**DOI:** 10.1186/s12936-020-3120-4

**Published:** 2020-01-22

**Authors:** Deepali Savargaonkar, Manoj Kumar Das, Amar Verma, Jeevan K. Mitra, C. P. Yadav, Bina Srivastava, Anupkumar R. Anvikar, Neena Valecha

**Affiliations:** 1 National Institute of Malaria Research, Sector 8, Dwarka, New Delhi 110077 India; 2 National Institute of Malaria Research, Field Unit, Ranchi, Jharkhand India; 3 Rajendra Institute of Medical Sciences, Ranchi, Jharkhand India

**Keywords:** Severe malaria, Injection artesunate, Delayed haemolysis, Haemoglobin, Lactate dehydrogenase

## Abstract

**Background:**

Parenteral artesunate is the treatment of choice for severe malaria. It is safe, efficacious and well tolerated anti-malarial. However, delayed haemolysis has been reported in travellers, non-immune individuals and in African children.

**Methods:**

A prospective, observational study was carried out in admitted severe malaria patients receiving parenteral artesunate. The patients were followed up until day 28 for monitoring clinical as well as laboratory parameters for haemolytic anaemia.

**Results:**

Twenty-four patients with severe malaria receiving injection artesunate were enrolled in the study. Post-artesunate delayed haemolysis following parenteral artesunate therapy was observed in three of 24 patients (12.5%, 95% confidence interval 4.5–31.2%). Haemolysis was observed in two more patients possibly due to other reasons. The haemoglobin fall ranged from 13.6 to 38.3% from day 7 to day 28 in these patients.

**Conclusion:**

The possibility of delayed haemolysis should be considered while treating the severe malaria patients with parenteral artesunate. The study highlights the need for further studies in different epidemiological settings.

## Background

Malaria is an infectious disease caused by *Plasmodium* parasites and transmitted by female *Anopheles* mosquitoes. Globally the estimated burden of malaria cases was 219 million cases while estimated malaria deaths were 435,000 in 2017 [1].

Globally the estimated annual incidence of severe malaria is reported to be two million [2]. India is standing on the horizon of malaria elimination era. Recent efforts have shown drastic decline in malaria cases and deaths in India [3]. Severe manifestations are reported in both vivax and falciparum malaria [4, 5]. Early diagnosis and timely and complete treatment is the key to malaria control, failing which, the disease can turn severe.

The World Health Organization (WHO) recommends parenteral artesunate for the treatment of severe malaria [6]. Artesunate rapidly clears parasites from blood. Studies have reported reduced risk of death in children as well as in adults receiving artesunate compared to those receiving quinine [7–9]. The occurrence of post-treatment hypoglycaemia is also shown to be less frequent with artesunate compared to quinine [7, 8]. However, occurrence of delayed haemolysis has been reported after injection artesunate in travellers in Europe [10, 11] as well as children under 5 years of age in Africa, including the Democratic Republic of Congo, Gabon and Ghana [12, 13]. A retrospective study carried out in Germany showed the occurrence of delayed haemolysis with artesunate but not with quinine [14]. Most of the data generated is in non-immune travellers and is mainly in the form of case reports and retrospective studies. There are various hypotheses for the mechanisms of haemolytic anaemia. Hence, the WHO recommends to carry out prospective studies to define frequency, magnitude and time course of delayed haemolysis [15].

A prospective study was carried out among severe malaria patients receiving parenteral artesunate to study its association with delayed haemolysis by assessing the haematological parameters.

## Methods

This prospective, observational study was carried out in severe malaria patients admitted to Rajendra Institute of Medical Sciences, Ranchi, a tertiary hospital in Jharkhand state. Patients were enrolled through June 2017 to January 2018. Severe malaria was defined as per the WHO criteria [16]. However, for definition of hyperparasitaemia, we used the cut off as 100,000 parasites/μl since India is a low endemic country [17].

All the clinically suspected and confirmed cases of malaria by microscopy and/or rapid diagnostic test (RDT), of all age groups between 6 months to 60 years, with severe manifestations and treated with parenteral artesunate, were included in the study after obtaining written informed consent/assent. Patients with known pregnancy, history of allergy to artesunate were excluded from the study.

### Data collection

Patient demographic information, history of allergy to any anti-malarial, history of any anti-malarial intake for the current episode of malaria, initial malaria diagnosis, signs and symptoms, anti-malarial treatment administered and any blood transfusion given were recorded.

### Laboratory procedures

Malaria diagnosis was performed using microscopy and RDT. Thick and thin blood films were prepared on the same slide, stained with Giemsa and examined under compound microscope. Parasite counting was done on thick films against 200 white blood cells (WBC). Density was calculated assuming 8000 WBC/μl of blood. Slides were declared negative if no parasites were detected in 100 high-power fields. Smears were collected at each follow up visit and examined for presence of parasite. RDT (SD Bio Line malaria Ag Pf/Pv, Standard Diagnostics, Inc., Republic of Korea) was also used for diagnosis. The test was performed as per the manufacturer’s instructions. Fever cases tested positive by RDT were also enrolled in the study.

From each enrolled case of severe malaria, 5 ml of blood was collected for haematological and biochemistry investigations. Haemoglobin, total leucocyte count (TLC), differential leucocyte count (DLC), reticulocyte count (%), screening for haemoglobin S, qualitative assay for glucose-6 phosphate dehydrogenase (G6PD), lactate dehydrogenase (LDH) and total bilirubin were performed on the day of enrollment.

Haemoglobin, TLC, DLC, reticulocyte count (%) were performed using haematology analyzer, screening for haemoglobin S was done by using solubility test [18], G6PD test was performed by using MBK G6PD qualitative assay, Lactate dehydrogenase and total bilirubin were performed by using biochemistry analyzer. Haemoglobin, reticulocyte count % and LDH were performed on all the follow up days also.

### Treatment

Treatment was given as per the Indian National Drug Policy on Malaria. Intravenous artesunate 2.4 mg/kg stat and then at 12 h, 24 h, and then once a day till patient can accept orally. This was followed by full course of oral ACT (artesunate + sulfadoxine–pyrimethamine in 19 and artemether–lumefantrine in 5 patients) [19].

Follow up was performed weekly up to day 28 and clinical assessment and laboratory investigations were carried out at each visit. Patients were asked for symptoms suggestive of haemolysis like presence of red coloured urine, abdominal pain and weakness. Delayed haemolysis was considered if there was > 10% fall in haemoglobin level along with increase in LDH concentration with or without rise in reticulocyte count % between day 7 to day 28 after initiation of injectable artesunate. In patients with haemolysis, the treatment was given with oral haematinics or blood transfusion as per the discretion of treating physician after assessing clinical condition.

## Results

A total of 32 patients were enrolled in the study. Of these, 24 were confirmed as *P. falciparum* positive by laboratory diagnosis, while eight were clinically suspected. The results of only confirmed malaria patients have been presented. Of the 24 patients, 10 were diagnosed by microscopy alone, five by RDT alone, and nine by both microscopy and RDT. The parasite count ranged from 64 parasites/µl to 2,45,000/µl. Four patients had parasitaemia of more than 1,00,000/µl. The enrolled patients had severe malaria with common presentations being severe anaemia, impaired consciousness and respiratory distress (Table 1).

**Table 1:** Baseline demographic and clinical characteristics of laboratory confirmed enrolled malaria patients (n = 24)

Gender	
Male	11 (45.83%)
Female	13 (54.16%)
Age	
Children (1–17 years)	21 (87.5%)
Adult (18 years and above)	3 (12.5%)
History of malaria and treatment taken	
History of treatment before reporting to study hospital	11 (45.83%)
History of malaria in last 6 months	2 (8.33%)
Clinical findings	
Palpable splenomegaly	10 (41.66%)
Palpable hepatomegaly	11 (45.83%)
Malaria diagnosis	
Positive by microscopy	10 (41.7%)
Positive by both microscopy RDT	9 (37.5%)
Positive by RDT	5 (20.8%)
Hemoglobin level	
Range of Hb level on day 0	4.9–13.1 g/dl
Severe malaria criteria on day 0	
Severe anemia	8 (33.33%)
Impaired consciousness	8 (33.33%)
Respiratory distress- SPO2 < 92% on room air with any signs of respiratory rate > 30/min or presence of crepitations on auscultation	6 (25%)
Hyperparasitemia	4(16.66%)
Jaundice- plasma or serum Bilirubin > 3 mg/dl	3 (12.5%)
Shock with systolic blood pressure < 70 mm Hg in children and < 80 mm Hg in adults	2 (8.33%)
Multiple convulsions	1 (4.16%)
Renal impairment—plasma or serum creatinine > 3 mg/dl	1 (4.16%)
Prostration	1(4.16%)

Figures in parentheses indicate percentage

Eleven out of 24 (46%) enrolled patients gave history of receiving treatment for fever before reporting to the study hospital. However, no treatment record was available with the patients.

The enrolled patients were followed up weekly until day 28. At least three follow ups could be performed in 18 patients. Two follow ups were performed in one patient; one follow up could be performed in three patients. Follow up could not be performed in two patients. Some of the follow up visits could not be performed due to reasons like long distance from the hospital since it is a tertiary care hospital, patients becoming free from symptoms, non-availability of the patients at their residence during active follow-up.

**Fig. 1:**
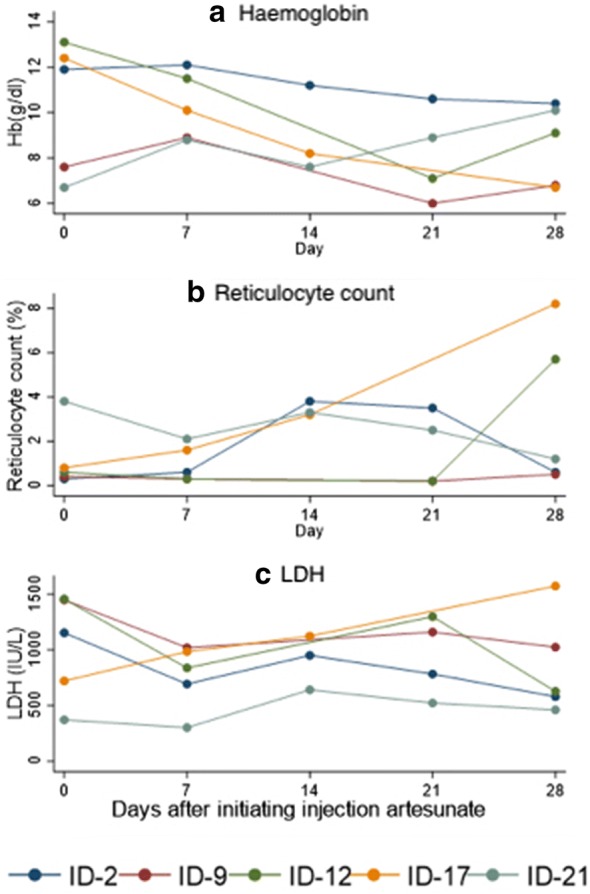
Haemoglobin levels (**a**), Reticulocyte count in % (**b**) and LDH levels (**c**) over time in laboratory confirmed malaria patients with haemolysis

During follow-up from day 7 to day 28, laboratory parameters like haemoglobin, LDH and reticulocyte count were monitored. Haemolysis was observed in 5 out of 24 patients with haemoglobin fall of more than 10% (Fig. 1, Table 2). None of these five patients had hyperparasitaemia. Among these 5, three patients (12.5%, 95% confidence interval 4.5% to 31.2%) also showed rise in LDH and reticulocyte percent followed by recovery. Thus, delayed haemolysis could be attributed to artesunate in these patients. Among the remaining 2, one patient had renal involvement. Another patient had persistent haemolysis with haemoglobin fall and rising LDH and reticulocyte percent until day 28. He also received parenteral ceftriaxone during hospitalization.

**Table 2:** Characteristics of the patients with haemolysis

Patient ID	Age in years/gender	Baseline parasitemia (parasites/µl)	Haemoglobin g/dl	LDH levels (IU/L)	Reticulocyte %	Total dose of intravenous artesunate (mg/kg)	Blood transfusion for hemolysis	Possible reason for haemolysis
2	7/F	63,485	Base line: 11.9 Day 7: 12.1 Minimum Hb: 10.4	Day 7: 694 Highest level (Day 14): 951	Day 7: 0.6 Highest level (Day 14): 3.8	14	None	Post artesunate delayed haemolysis
9	13/M	143	Base line 7.6 Day 7: − 8.9 Minimum Hb: 6	Day 7: 1021 Highest level (Day 21): 1161	Day 7: 0.3 Highest level (Day 28): 0.5	14	None	Renal involvement
12	2.4/M	2496	Base line 13.1 Day 7: − 11.5 Minimum Hb: 7.1	Day 7: 839 Highest level (Day 21): 1300	Day 7: 0.3 Highest level (Day 28): 5.7	14	Day-21	Post artesunate delayed haemolysis
17	13/M	394	Base line: 12.4 Day 7: 10.1 Minimum Hb: 6.7	Day 7: 985 Highest level (Day 28):1575	Day 7: 1.6 Highest level (Day 28): 8.2	12	None	Intravenous ceftriaxone
21	2/M	8748	Base line: 6.7 Day 7: 8.8 Minimum Hb: 7.6	Day 7: 302 Highest level (Day 14): 642	Day 7: 2.1 Highest level (Day 14): 3.3	15	None	Post artesunate delayed haemolysis

The fall of haemoglobin from day 7 to day 28 ranged from 13.6% to 38.3%. All these patients were G6PD nondeficient and sickling test was negative. Two patterns of haemoglobin fall were observed: three patients had initial rise by day 7 followed by fall, while two patients experienced continuous fall (Fig. 1). Packed RBC transfusion was indicated during follow up in one of these five patients. Other four patients were managed with oral haematinics like iron and folic acid tablets. Four of the five patients were discharged in stable condition and blood smears were negative for malaria parasites at all the follow-up visits. One patient had deranged renal functions on admission as well as on discharge on day 7. The patient left against medical advice.

## Discussion

Anaemia is a common feature of malaria. However the delayed haemolytic anaemia after administration of artesunate occurs when there is clearance of parasites from blood and resolution of malaria related symptoms. Post artesunate delayed haemolysis was observed in 3 patients during follow up from day 7 to day 28. A study has reported delayed haemolysis with fall in haemoglobin and rise in LDH from day 15 to day 32 after first dose of injection artesunate [10].

Few studies from Africa, Germany and Spain observed delayed haemolysis as a frequent complication in hyperparasitaemic severe malaria patients administered with injectable artesunate [13, 20, 21]. However, another study conducted in Africa reported that delayed haemolysis was uncommon despite the patients having hyperparasitaemia [22]. Delayed haemolysis was not observed among the hyperparasitaemic patients in the present study. Thus, the risk of haemolysis might be dependent on malaria endemicity of the area. Although a case report of post artesunate delayed haemolysis in hyperparasitaemic patient has been published from India [23], this is the first systematic study that monitored post artesunate delayed haemolysis by evaluating various parameters.

For treatment of severe malaria, Injection artesunate for at least 24 h and full course of ACT once the patient can take orally is recommended by the WHO as well as Indian National Drug policy [6, 19]. The post-artesunate delayed haemolysis has also been reported following oral ACT for uncomplicated malaria. However, it was milder compared to haemolysis with parenteral artesunate [24], rectal artesunate and intramuscular artemether in severe malaria patients [14, 25].

All the patients were treated with parenteral artesunate followed by ACT. Thus, delayed haemolysis may be due to parenteral artesunate or combined effect of both. Two of the three patients with delayed haemolysis recovered with oral haematinics while blood transfusion was needed in one patient. Few studies have reported need of blood transfusion in patients with delayed haemolysis, however no deaths have been reported due to this phenomenon [11, 20]. Few case reports suggest immune mechanism associated with post artesunate delayed haemolysis and patients responded to corticosteroid therapy [23, 26, 27]. Other studies mention pitting of RBCs as the reason for delayed haemolysis where the parasites in the RBCs are killed by artesunate and removed in spleen. The once infected RBCs remain in the circulation with reduced lifespan [28]. The early concentration of once infected erythrocytes is reported to be a marker to predict delayed haemolysis [29]. The evidence for pitting as the mechanism for delayed haemolysis seems stronger than immunological mechanisms [15, 30].

For assessing haemolysis, various markers like haemoglobin, LDH, reticulocyte percent, haptoglobin are evaluated [10, 13]. In this study, haemoglobin, LDH and reticulocyte percent were evaluated. The reticulocyte percent increased by day 21 in two patients and by day 28 in one patient (ID 12). This patient received blood transfusion on day 0 and day 21. This could be the reason for delay in increase in reticulocyte percent. It has been reported that blood transfusion may be responsible for low reticulocyte count [31].

In two patients, there could be other reasons for haemolysis apart from artesunate. There was one patient (ID 9) who had haemolysis but had no rise in reticulocyte percent by day 28. He had deranged renal functions. Low reticulocyte counts have been reported in patients with renal involvement and delayed haemolysis [32, 33]. The possible reason could be low erythropoietic production due to renal involvement [34].

One patient (ID 17) experienced persistent haemolysis with haemoglobin fall along with increase in reticulocyte percent and LDH until day 28. He also received parenteral ceftriaxone as empirical therapy. The continuous fall may be attributed to drug induced immune haemolytic anemia. Drug induced haemolysis due to ceftriaxone has been reported in patients with falciparum malaria [35].

All the patients with delayed haemolysis were discharged in stable condition and their blood smears were negative for malaria parasites at all the follow-up visits. In areas where population has low level of haemoglobin, delayed haemolysis can be fatal and need to be monitored closely to provide timely treatment. Few of the enrolled patients had received treatment before reporting to study hospital. The chances of patients administered with anti-malarials by private practitioners before reporting to the study hospital cannot be ruled out, though records of medicines or of prescription were not available. The possibility of delayed haemolysis following injectable artesunate may be kept in mind. There is need to conduct studies in different epidemiological settings in larger sample. However, the clinicians should be made aware of the delayed haemolysis and need of monitoring for haemoglobin fall for 4 weeks.

## Conclusion

In view of delayed haemolysis in 12.5% patients of severe malaria receiving parenteral artesunate, there is need to monitor the haemolytic parameters. Further studies are required in large number of patients and in different epidemiological settings.

## Data Availability

The authors declare that the data of the study is available in electronic form. The datasets used and/or analysed during the current study are available from the corresponding author on reasonable request.

## Abbreviations

ACT: artesunate based combination therapy
DLC: differential leucocyte count
G6PD: glucose 6 phosphate dehydrogenase
Hb: haemoglobin
IU: international unit
LDH: lactate dehydrogenase
Pf: *P. falciparum*
Pv: *P. vivax*
RBC: red blood cell
RDT: rapid diagnostic test
TLC: total leucocyte count
WBC: white blood cell
WHO: World Health Organization
